# Standardised colour-coded compartmentalised syringe trays improve anaesthetic medication visual search and mitigate cognitive load^☆^

**DOI:** 10.1016/j.bja.2022.11.012

**Published:** 2023-01-10

**Authors:** Victoria Laxton, Frances A. Maratos, David W. Hewson, Andrew Baird, Edward J.N. Stupple

**Affiliations:** 1 College of Health, Psychology and Social Care, University of Derby, Derby, UK; 2 TRL, Wokingham, UK; 3 Academic Unit of Injury, Recovery and Inflammation Sciences, School of Medicine, University of Nottingham, Nottingham, UK; 4 Department of Anaesthesia and Critical Care, Queen's Medical Centre, Nottingham University Hospitals NHS Trust, Nottingham, UK

**Keywords:** cognitive load, colour-coding, eye-tracking, medication error, syringe trays, visual search

## Abstract

**Background:**

Anaesthetic procedures are complex and subject to human error. Interventions to alleviate medication errors include organised syringe storage trays, but no standardised methods for drug storage have yet been widely implemented.

**Methods:**

We used experimental psychology methods to explore the potential benefits of colour-coded compartmentalised trays compared with conventional trays in a visual search task. We hypothesised that colour-coded compartmentalised trays would reduce search time and improve error detection for both behavioural and eye-movement responses. We recruited 40 volunteers to identify syringe errors presented in pre-loaded trays for 16 trials in total: 12 error present and four error absent, with eight trials presented for each tray type.

**Results:**

Errors were detected faster when presented in the colour-coded compartmentalised trays than in conventional trays (11.1 s *vs* 13.0 s, respectively; *P*=0.026). This finding was replicated for correct responses for error-absent trays (13.3 s *vs* 17.4 s, respectively; *P*=0.001) and in the verification time of error-absent trays (13.1 s *vs* 17.2 s, respectively; *P*=0.001). On error trials, eye-tracking measures revealed more fixations on the drug error for colour-coded compartmentalised trays (5.3 *vs* 4.3, respectively; *P*<0.001), whilst more fixations on the drug lists for conventional trays (8.3 *vs* 7.1, respectively; *P*=0.010). On error-absent trials, participants spent longer fixating on the conventional trials (7.2 s *vs* 5.6 s, respectively; *P*=0.002).

**Conclusions:**

Colour-coded compartmentalisation enhanced visual search efficacy of pre-loaded trays. Reduced fixations and fixation times for the loaded tray were shown for colour-coded compartmentalised trays, indicating a reduction in cognitive load. Overall, colour-coded compartmentalised trays were associated with significant performance improvements when compared with conventional trays.

**Editor's key points**

• Anaesthetic medications are administered under dynamic and distracting conditions that lead to considerable cognitive demand that can increase medication errors.
• We hypothesised that colour-coded compartmentalised trays reduce search time and improve error detection in correct syringe selection.
• In a simulation study of 40 volunteers, colour-coding enhanced visual search efficacy by reducing fixations and fixation times, indicating a reduction in cognitive load.
• Colour-coded compartmentalised trays were associated with performance improvements compared with conventional trays, which now requires clinical validation.

Drugs for anaesthesia and sedation are prepared and administered in complex clinical environments under dynamic and distracting conditions. This leads to considerable cognitive demand that can decrease task performance and increase medication errors.^1^ Drug-related errors occur in one in 133 anaesthetics,^2^ and 2% of drug error cases result in complications, serious harm, or death.^3^ Syringe swaps and drug misidentifications are common anaesthetic drug errors, appearing in 40–70% of incident reports.^4^^,^^5^ It is therefore important to understand how these errors can be reduced.

Improving workspace organisation might mitigate some adverse cognitive load in busy clinical settings and improve patient safety through standardisation. The effects of demanding clinical environments on patient safety in relation to drug administration can be measured with several outcomes, such as frequency of drug error or indices of cognitive demand and efficiency.^6^, ^7^, ^8^

Initiatives, such as colour-coded drug labels, electronic drug checking, and workspace redesign,^9^, ^10^, ^11^, ^12^, ^13^, ^14^, ^15^ have been introduced to improve error prevention and can mitigate some routes to error. In a feasibility study, anaesthetists reported that a compartmentalised, colour-coded drug tray facilitated improved syringe identification performance compared with tasks undertaken with non-compartmentalised non-colour-coded ‘conventional’ trays.^16^

Effects of cognitive load and overload have been explored through eye-movement tracking.^17^^,^^18^ Cognitive overload leads to restricted eye movements,^19^ fewer attentive eye movements, fewer fixations,^20^ longer fixation duration, and longer verification times.^21^ Such effects have been demonstrated in many real-world tasks, such as automobile driving,^22^^,^^23^ aviation, and water safety.^24^ In surgical settings, eye-tracking has been used to explore team dynamics during simulated training and operations.^25^^,^^26^

We used eye-tracking metrics and behavioural data to test the potential performance benefits of colour-coded compartmentalised syringe trays compared with conventional trays in relation to visual search activities. We hypothesised that (i) colour-coded trays improve efficiency of drug error detection, including faster responses for both error-present and error-absent trays compared with conventional trays; (ii) conventional trays increase cognitive load, evidenced by increased fixations to a list of drugs that should be present in the tray; and (iii) colour-coded trays have shorter fixation durations to drug errors and shorter verification times for error presence compared with conventional trays.

## Methods

This study protocol received ethical approval from the 10.13039/100010025University of Derby (reference: ETH2122-0251; granted June 15, 2021) and 10.13039/100005622Health Research Authority (reference: 21/HRA/1087; granted May 21, 2021). Consent was obtained from all participants *via* an online form hosted by Qualtrics (Provo, UT, USA). The 10.13039/100010025University of Derby served as the study sponsor. The study was conducted at two sites: University of Derby Clinical Skills Suite, Derby, UK, and Nottingham University Hospitals NHS Trust Trent Simulation and Clinical Skills Centre, Nottingham, UK.

### Participants

Participants were registered operating department practitioners or operating department practitioners enrolled in an apprenticeship programme. Participants were recruited at the University of Derby and through posters at the testing locations. All participants had experience in the operating theatre and anaesthetic environment and were aware of the specific drugs used in anaesthetic procedures and typical errors that might occur.

Using a two-sided matched-pairs *t*-test, a total of 39 participants would be required to detect a medium effect size (*d*=0.6) for drug-detection errors as a function of tray type (colour-coded *vs* conventional) with β=0.95 and α=0.05. Such a sample size is consistent with previous literature that has explored eye movements in real-world (*in situ*) settings with specialised populations.^24^^,^^26^, ^27^, ^28^

### Stimuli and apparatus

#### Tray designs

**Fig 1:**
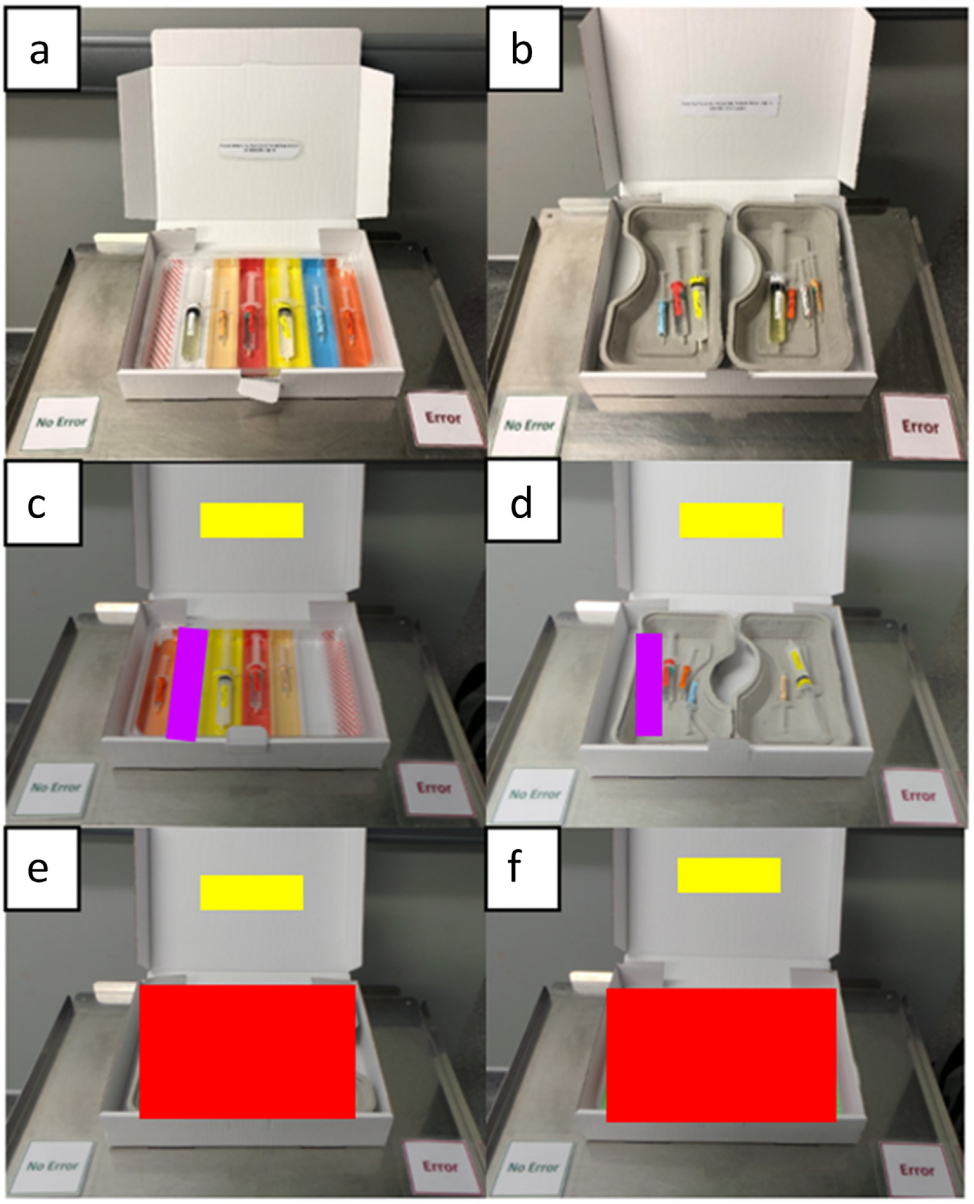
Snapshot images of (a) colour-coded trays and (b) conventional trays. An ‘additional drug’ error in (c) colour-coded tray and (d) conventional tray. In (c) and (d), the errors and drug lists are framed by an area of interest (AOI window), with the tray error AOI highlighted in purple and the drug list AOI highlighted in yellow. In colour-coded and conventional error-absent trays, (e) and (f), respectively, the drug list AOI is framed in yellow and the full tray AOI (covering the entire tray) in red.

The tray used for the study intervention was a compartmentalised, colour-coded tray organised by drug class matching the ISO 26825:2020 international colour-coded labelling system^29^ (Rainbow Trays™; UVAMED, Loughborough, UK; Fig 1a).

The conventional tray used for the study control was a single compartmented, grey, multipurpose paper mulch tray found in many hospitals (Fig 1b).

### Error detection task

Colour-coded and conventional trays were loaded with typical anaesthetic drugs for eight pre-specified anaesthetic scenarios (Table 1). Three drug-error categories were incorporated into the scenarios: additional drug, missing label, and allergy risk, alongside a no-error condition. There were two scenarios for each category. Scenarios were matched for each tray condition and presented in a random order. In total, there were 16 loaded trays and 16 anaesthetic scenarios. Of the 16 trials, 75% contained errors.

**Table 1:** Example of the anaesthetic scenario and drug list. Scenarios were counterbalanced between tray types, with changes in patient characteristics to avoid scenario repetition.

Tray condition	Surgery	Scenario 1	Scenario 2	Drug list	Error
Colour-coded compartment Tray 1	Low back pain surgery	35-yr-old female; good general health; no known conditions, allergies, or medications; BMI in a healthy range	41-yr-old male; healthy weight range; no known conditions, allergies, or medications	Propofol	Additional neuromuscular blocking drug
				Midazolam	
				Atracurium	
				Fentanyl	
				Ondansetron	
				Ketamine	
Colour-coded compartment Tray 2	Pacemaker implantation	78-yr-old male classed as obese; history of high blood pressure and heart complications	72-yr-old female classed as obese; history of heart complications and has diabetes mellitus	Propofol	Additional opioid
				Midazolam	
				Rocuronium	
				Fentanyl	
				Droperidol	
				Cefuroxime	
				Neostigmine	
Conventional Tray 1	Low back pain surgery	41-yr-old female; healthy weight range; no known conditions, allergies, or medications	35-yr-old male; good general health; no known conditions, allergies, or medications; BMI in a healthy range	Propofol	Additional neuromuscular blocking drug
				Midazolam	
				Atracurium	
				Fentanyl	
				Ondansetron	
				Ketamine	
Conventional Tray 2	Pacemaker implantation	72-yr-old male classed as obese; history of heart complications and has diabetes	78-yr-old female classed as obese; history of high blood pressure and heart complications	Propofol	Additional opioid
				Midazolam	
				Rocuronium	
				Fentanyl	
				Droperidol	
				Cefuroxime	
				Neostigmine	
Colour-coded compartment Tray 1	Tonsillectomy	33-yr-old male; BMI in healthy range; good general health; penicillin allergy	24-yr-old female; BMI in healthy range; good general health; penicillin allergy	Propofol	None
				Midazolam	
				Atracurium	
				Fentanyl	
				Ondansetron	
				Teicoplanin	
Conventional Tray 1	Emergency appendectomy	22-yr-old female; good general health; active abdominal sepsis; no known conditions or allergies	19-yr-old male; good general health; active abdominal sepsis; no known conditions or allergies	Propofol	None
				Midazolam	
				Atracurium	
				Morphine	
				Ondansetron	
				Co-amoxiclav	

Loaded trays were placed in a plain white box to avoid participants seeing an earlier view of the loaded tray. The scenario and drug list were then placed on the front of the box. The scenarios were counterbalanced between participants (see Table 1 and Fig 1 for a detailed example).

Laminated ‘error’ and ‘no error’ signs were placed adjacent to the box, with the error sign positioned to the left and the no error sign positioned to the right. The participants determined if an error was present in the loaded tray and pointed to the relevant sign to indicate their responses. Both pointing and verbal responses were used to record participant accuracy and response times.

### Outcome measures

#### Accuracy and response time measures

Error detection performance was measured with response accuracy and response times. Response times were calculated from when the box was fully opened until a pointing action or verbalisation was observed. Responses were noted as accurate if an ‘error’ response was made to an error-present trial or a ‘no-error’ response to an error-absent trial.

#### Eye-movement measures

Eye movements and responses were recorded using Tobii Pro Glasses 3 (Tobii, Stockholm, Sweden). Video footage from the glasses was analysed in Tobii Pro Lab and areas of interest (AOI), as defined in Figure 1c–f.

We set a sampling rate of 50 Hz and accuracy of 0.6°. There were four eye-tracking sensors, two per eye. The scene camera's field of view captured 95° horizontally and 63° vertically. A one-point ‘look and fixate’ calibration procedure was used. The following measures were analysed:

(i) *Time to first fixation on the error:* on error-present trials, the first fixation to the error was calculated from when the box lid was fully open (the first time the error is presented) to the first fixation on the error AOI.
(ii) *Verification times:* the ‘time to verify’ if an error was present, or otherwise, was measured from the initial fixation on the AOI to when the button press/verbal confirmation was made (i.e. error or no error). Here, data from both error-present and error-absent trials were explored.
(iii) *Fixation count:* the number of fixations made to tray error AOI or the drug list AOI. For error-absent trials, only drug list AOI existed and could be analysed.
(iv) *Average fixation duration:* the average times participants fixated on the tray AOI and the list AOI were analysed to explore both error-present and error-absent trials.

#### Cognitive load

To explore the effects of cognitive load as a function of tray type, the number of fixations made to the AOI and average fixation durations to the AOI were analysed. Error-present trials were explored with a comparison between tray type (colour-coded *vs* conventional) and AOI fixations (tray-error AOI *vs* drug-list AOI). In error-absent trials, the same analyses could be processed for average fixation duration (tray AOI *vs* drug-list AOI). For fixation count, only a comparison between drug list AOI fixations for both tray types could be examined.

#### Error-absent correct rejections

Responses and verification times were used to explore the impact of tray type on the correct rejection of error-absent trials.

### Procedure

Consenting participants attended a testing session in a quiet room at either the University of Derby Clinical Skills Suite or the Nottingham University Hospitals Trent Simulation Centre. The eye tracker was fitted and calibrated, and participants were given a practice trial. Once participants understood the task, the experiment, consisting of 16 trials, was conducted. All participants were fully debriefed on the general purpose of the research upon completion.

### Statistical analysis plan

Error-present and error-absent trials were analysed in relation to the accuracy of responses, response times, time to first fixate on an error, and verification time by paired *t*-tests using SPSS (IBM, Armonk, NY, USA). Fixation count and average fixation duration for the error present trials were explored with a tray (colour-coded *vs* conventional) × AOI (tray error AOI *vs* drug list AOI) repeated-measures analysis of variance (anova). Average fixation duration for the error-absent trials was explored with a tray (colour-coded *vs* conventional) × AOI (tray AOI *vs* drug list AOI) repeated-measures anova, whereas a *t*-test was used to explore fixation count to the drug list AOI by tray type. For error-absent trays, the entire tray was the AOI, as there was no item to identify; therefore, fixation count differences were captured by the fixations to the drugs list only.

## Results

Between July 28, 2021 and November 30, 2021, we recruited 40 participants (mean age 36 yr; standard deviation [sd]=9.4) into the study. All participants had experience as operating theatre staff and were aware of anaesthetic drugs used and errors that might occur in operating theatre environments.

Data screening resulted in removal of three participants because of failure to complete the task as expected (*n*=1), technical failure in the eye-tracking glasses recording system (*n*=1), and a tracking ratio <30% (*n*=1). Tracking ratios indicate the proportion of time that the eye tracker recorded point of gaze coordinates over the entire task. All remaining participants (*n*=37) had a good tracking ratio (average 95%) and completed the study.

### Error-present trial analysis

Accuracy scores, response time to errors, time to first fixate an error, and verification time were all explored for error-present trials. For the accuracy data, responses were considered correct if a drug error was correctly identified. The mean [sd] difference between colour-coded and conventional trays in correct error identification was not significant (89 [31]% *vs* 85 [36]%; *P*=0.173).

Correctly identified errors were detected faster for colour-coded than conventional trays (11 [7.6] s *vs* 13 [8.3] s; *t* [36]=–2.32; *P*=0.026; Cohen's *d*=–0.38). However, there was no difference between tray types in the time to first fixate the error (2.8 [2.7] *vs* 3.4 [1.3] s; *P*=0.167) nor for error verification time (8.8 [8.8] *vs* 9.6 [7.9] s; *P*=0.261).

### Error-absent trial analysis

Accuracy and response times were explored for error-absent trials. For correct rejections (the observation that no error was present), the difference between colour-coded and conventional trays was not significant (94 [23]% *vs* 96 [20]%; *P*=0.711). However, the difference between response times for correct rejections was faster for colour-coded than for conventional trays (13.3 [7.0] s *vs* 17.4 [10.1] s; *t* [36]=–3.63; *P*=0.001; Cohen's *d*=–0.60). The verification time for error-absent trials also demonstrated that colour-coded trays were processed faster than conventional trays (13.1 [7.0] s *vs* 17.2 [10.1] s; *t* [36]=–3.58; *P*=0.001; Cohen's *d*=–0.60).

### Fixation count on error-present trials

**Fig 2:**
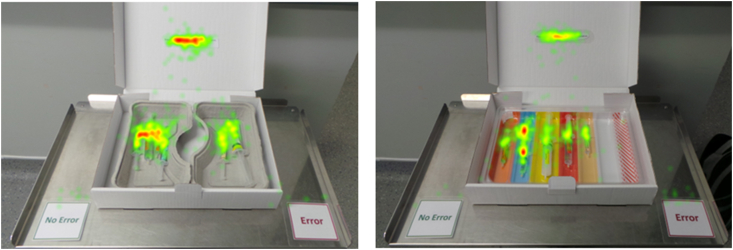
Heat maps of participant fixations across the two tray types in an error trial example. Red indicates more fixations to that area. There were greater fixations to the drug list for conventional trays (left) compared with greater fixations on the drug error for colour-coded trays.

Error trials were explored in a tray (colour-coded *vs* conventional) × AOI (tray error AOI *vs* list AOI) repeated-measures anova (Table 2). The main effect of tray was not significant (*P*=0.771). There was, however, a main effect of AOI (*F* [36]=26.45; mean square error [MSe]=11.65; *P*<0.001; $\eta_{p}^{2}$ =0.42), with the drug list AOI receiving more fixations than the tray error AOI (7.7 [3.6] *vs* 4.8 [1.5], respectively). There was also an interaction between tray type and AOI (*F* [36]=21.27; MSe=2.32; *P*<0.001; $\eta_{p}^{2}$ =0.37). *Post hoc* Bonferroni-corrected *t*-tests showed significantly more fixations on the error AOI for the colour-coded trays compared with the conventional trays (*t* [36]=4.07; *P*<0.001; Cohen's *d*=0.67) and significantly fewer fixations on the drug list for the colour-coded trays than the drug list for the conventional trays (*t* [36]=–2.48; *P*=0.018; Cohen's *d*=–0.41) (Table 2; Fig 2).

**Table 2:** Mean fixation count for the drug error AOI and the drug list AOI as a function of tray type (standard deviation in parentheses). AOI, area of interest.

AOI	Tray type		AOI mean
	Colour-coded	Conventional	
Tray error	5.3 (1.8)	4.3 (1.6)	4.8 (1.5)
Drug list	7.1 (3.6)	8.3 (4.1)	7.7 (3.6)
Tray type mean	6.2 (2.2)	6.3 (2.5)

### Fixation count on error-absent trials

Analysis of the drug list AOI for error-absent trials revealed a significant difference with more fixations on the conventional tray drug list than the colour-coded tray drug list (10.4 [6.7] *vs* 8.0 [5.4], respectively; *t* [36]=–2.74; *P*=0.010; Cohen's *d*=–0.45).

### Average fixation duration for error-present trials

For error trials, average fixation duration was explored in a tray (colour-coded *vs* conventional) × AOI (tray error AOI *vs* drug list AOI) repeated-measures anova. The main effect of tray was not significant (*P*=0.603). There was, however, a main effect of AOI (*F* [36]=84.32; MSe=1.36; *P*<0.001; $\eta_{p}^{2}$ =0.70), with the drug list AOI receiving longer fixations than the tray error AOI (3.8 [1.5] s *vs* 2.1 [0.7] s). There was an interaction between tray type and AOI window (*F* [36]=4.51; MSe=0.50; *P*=0.005; $\eta_{p}^{2}$ =0.20) (Table 3). *Post hoc* Bonferroni-corrected *t*-tests showed that average fixation durations did not differ as a function of tray type for tray error AOI (*P*=0.058) or drug list AOI (*P*=0.085).

**Table 3:** Mean fixation duration for drug error AOI and drug list AOI as a function of tray type. AOI, area of interest; sd, standard deviation.

AOI	Tray type		AOI mean (sd) (s)
	Colour-coded (s)	Conventional (s)	
Tray error	2.2 (0.9)	1.9 (0.7)	2.0 (0.7)
List	3.6 (1.5)	4.0 (1.8)	3.8 (1.5)
Tray type mean (sd)	2.9 (1.1)	3.0 (1.1)	

### Average fixation duration for error-absent trials

A tray (colour-coded *vs* conventional) × AOI (full tray AOI *vs* drug list AOI) repeated-measures anova revealed a main effect of tray (*F* [36]=11.62; MSe=7.86; *P*=0.002; $\eta_{p}^{2}$ =0.24) and a main effect of AOI (*F* [36]=39.75; MSe=15.64; *P*<0.001; $\eta_{p}^{2}$ =0.53). For the main effect of tray, participants spent more time fixating on the full tray/drug list AOI for conventional trays compared with colour-coded trays (7.2 [4.0] s *vs* 5.7 [2.9] s, respectively). For the main effect of AOI, all participants spent longer viewing the tray AOI compared with the drug list AOI (8.5 [4.9] s *vs* 4.4 [2.0] s, respectively). The interaction effect between tray type and AOI was not significant (*P*=0.626).

## Discussion

The purpose of this study was to determine whether colour-coded compartmentalised syringe trays enhanced visual search performance compared with conventional trays. Analyses revealed colour-coded trays elicited faster error detection, acceptance, and verification for error-absent trials compared with conventional trays. There was evidence of cognitive load mitigation for colour-coded trays, with shorter and fewer fixations to the drug lists on error trials, and trays (i.e. full tray and drug list) on error-absent trials compared with conventional trays.

The advantages found for colour-coded trays were consistent with previous research.^14^^,^^16^ For colour-coded trays, there was no speed–accuracy trade-off, as accuracy was not negatively affected by faster responses. This accords with research showing similar advantages of colour-coding in search display.^30^ Cognitive-load benefits were also seen for colour-coded trays, with shorter and fewer fixations to the drug list on error trials and the tray *per se* on error-absent trials. This is consistent with prior research showing a reduced workload for colour-coding in other domains,^3^^,^^31^^,^^32^ in anaesthetic machines,^32^ and in studies showing fewer eye movements in high cognitive demand situations.^20^ Our results are consistent with the findings that colour-coding is a tool to aid detection, discrimination, and classification of stimuli by enabling more efficient encoding of information and faster comprehension.

The organisation of colour-coded trays could facilitate secondary checks from operating theatre staff, such as operating department practitioners. Secondary checking, either through human checks or electronic checks, is an additional safety layer to prevent drug errors, but it does not always happen because of time constraints, operating theatre pressures, and impracticalities.^33^^,^^34^ The clear layout of the colour-coded trays means staff can efficiently check if the right drugs for the intended surgery have been loaded into the tray and the correct drug has been selected to be administered. In this (admittedly artificial) task, most participants were able to perform a syringe drug list check for the colour-coded trays in <15 s. The data for colour-coded compartmentalised trays were consistent with enhanced visual attention and amelioration of cognitive processing bottlenecks.

One caveat is that syringes were neatly arranged in both tray conditions. Within operating theatres, syringes in conventional trays can be haphazardly arranged, as there are no compartments constraining syringe arrangement or movement within the tray. The design choice to arrange syringes neatly in both types of tray was made to ensure that all drug labels were equally readable in both conditions. Consequently, the relative benefit of compartmentalisation offered by colour-coded trays is arguably reduced by neatly arranging syringes in the conventional trays. Under more ecologically valid conditions, variation in syringe orientation within conventional trays is likely to increase task difficulty. Thus, there may be merit in future investigations for using a haphazard arrangement of syringes in conventional trays when comparing with colour-coded compartmentalised trays.

Future research should explore the effectiveness of colour-coded trays specifically with anaesthetists. Whilst this study demonstrated the benefits of the colour-coded trays for operating theatre staff and highlights the potential benefits for secondary checks by medical staff, it would be beneficial to explore whether anaesthetists obtain similar benefits as the primary user. This could be explored through a similar visual search task for error-present and error-absent trays, with a manipulation on cognitive load, to replicate the busy and mentally demanding conditions of the anaesthetist's role.

In conclusion, colour-coded compartmentalised trays demonstrated cognitive load and visual search performance advantages over conventional trays when identifying medication errors. This was evidenced by both faster behavioural responses and faster processing of the loaded tray. Colour-coded trays have the potential to provide both organisational and standardisation benefits in anaesthesia, enabling fast and efficient checking of a drug-loaded tray. These trays also demonstrate the potential for reduced cognitive load amongst their users.

## Authors' contributions

Study conception/design: all authors

Development of materials for the study: VL, FAM, DWH, EJNS

Data collection/analysis: VL

Writing of article: all authors

Revising of article: FAM

All authors revised the article for significant intellectual content, gave approval for the version to be published, and agree to be accountable for the work in ensuring that questions related to the accuracy or integrity of any part of the work are appropriately investigated and resolved.
